# ColiSeq: a multiplex amplicon assay that provides strain level resolution of *Escherichia coli* directly from clinical specimens

**DOI:** 10.1128/spectrum.04139-23

**Published:** 2024-04-23

**Authors:** Charles H. D. Williamson, Adam J. Vazquez, Amalee E. Nunnally, Kristen Kyger, Viacheslav Y. Fofanov, Tara N. Furstenau, Heidie M. Hornstra, Joel Terriquez, Paul Keim, Jason W. Sahl

**Affiliations:** 1 Pathogen and Microbiome Institute, Northern Arizona University, Flagstaff, Arizona, USA; 2 School of Informatics, Computing, and Cyber Systems, Northern Arizona University, Flagstaff, Arizona, USA; 3 Flagstaff Medical Center, Flagstaff, Arizona, USA; Michigan State University, East Lansing, Michigan, USA

**Keywords:** UTIs, genotyping, *E. coli*, amplicon sequencing

## Abstract

*Escherichia coli* is a diverse pathogen, causing a range of disease in humans, from self-limiting diarrhea to urinary tract infections (UTIs). Uropathogenic *E. coli* (UPEC) is the most frequently observed uropathogen in UTIs, a common disease in high-income countries, incurring billions of dollars yearly in treatment costs. Although *E. coli* is easily grown and identified in the clinical laboratory, genotyping the pathogen is more complicated, yet critical for reducing the incidence of disease. These goals can be achieved through whole-genome sequencing of *E. coli* isolates, but this approach is relatively slow and typically requires culturing the pathogen in the laboratory. To genotype *E. coli* rapidly and inexpensively directly from clinical samples, including but not limited to urine, we developed and validated a multiplex amplicon sequencing assay, called ColiSeq. The assay consists of targets designed for *E. coli* species confirmation, high resolution genotyping, and mixture deconvolution. To demonstrate its utility, we screened the ColiSeq assay against 230 clinical urine samples collected from a hospital system in Flagstaff, Arizona, USA. A limit of detection analysis demonstrated the ability of ColiSeq to identify *E. coli* at a concentration of ~2 genomic equivalent (GEs)/mL and to generate high-resolution genotyping at a concentration of 1 × 10^5^ GEs/mL. The results of this study suggest that ColiSeq could be a valuable method to understand the source of UPEC strains and guide infection mitigation efforts. As sequence-based diagnostics become accepted in the clinical laboratory, workflows such as ColiSeq will provide actionable information to improve patient outcomes.

**IMPORTANCE:**

Urinary tract infections (UTIs), caused primarily by *Escherichia coli*, create an enormous health care burden in the United States and other high-income countries. The early detection of *E. coli* from clinical samples, including urine, is important to target therapy and prevent further patient complications. Additionally, understanding the source of *E. coli* exposure will help with future mitigation efforts. In this study, we developed, tested, and validated an amplicon sequencing assay focused on direct detection of *E. coli* from urine. The resulting sequence data were demonstrated to provide strain level resolution of the pathogen, not only confirming the presence of *E. coli*, which can focus treatment efforts, but also providing data needed for source attribution and contact tracing. This assay will generate inexpensive, rapid, and reproducible data that can be deployed by public health agencies to track, diagnose, and potentially mitigate future UTIs caused by *E. coli*.

## INTRODUCTION

*Escherichia coli* is a highly versatile species of bacteria, serving as an essential member of the human gut microbiome (1), a model organism in biotechnology (2) and genetic research (3), and causing a range of disease in humans, including meningitis (4), hemolytic uremic syndrome (5), and urinary tract infections (UTIs) (6). In lower- and middle-income countries, diarrheagenic *E. coli* causes severe disease and excess mortality through inadequate sanitation, contaminated water sources, and limited access to healthcare (7). UTIs, including bladder infections, are the primary health risk of *E. coli* in high-income countries (8, 9), where uropathogenic *E. coli* (UPEC) is the primary uropathogen. In the United States, UTIs result in millions of healthcare visits annually and incur an economic burden well into the billions of dollars (10–12). Untreated or antimicrobial-resistant bladder infections can cause pyelonephritis, which can result in sepsis and death if not treated (13).

A key component to effective management and treatment of UTIs is diagnosis of the infective agent. Clinical labs rarely identify pathogen strains or genotypes and focus instead on rapid detection and phenotypic characterization so that treatment may proceed as soon as possible (14).The detection of UPEC in urine is typically focused on species identification using culture confirmation (15) or metabolite-based methods (16). More rarely, metagenomics (17) or whole-genome sequencing (WGS) (18) are applied which provide high-resolution genotyping but at a higher cost. Shotgun metagenomics requires expensive deep sequencing to overcome the high concentration of host background DNA, and WGS approaches often require culturing, which can substantially delay the time to answer needed for prompt interventions. Consequently, although clinical labs process large collections of samples, they typically are unable to provide the genotype information required to understand larger patterns of transmission, circulation, and coinfection (19, 20). Genotypic information could guide clinicians’ choices of antibiotic regimens or could help differentiate between recurrent and new infections.

Researchers have identified multiple, independent genotypes of UPEC associated with UTIs many of which fall within the B2 phylogroup (21), including the antibiotic-resistant clone, ST131 (22). A recent study used WGS-based methods to identify cases of zoonotic extraintestinal *E. coli* transfer (23), which illustrates the importance of obtaining strain level information from UTI samples to understand pathogen spread. There is limited information on the frequency of mixed infections, but it is important that health-care professionals are aware of the possible presence of co-infections of multidrug-resistant UPEC strains when treating patients due to the risk of life-threatening complications (24). In some clinical stool samples, mixtures of pathogenic variants (pathovars) or strains within pathovars have been observed (25). Methods have been developed to characterize mixed bacterial infections, including processing colony sweeps (26), single colony screens (27), and haplotype analyses (28). In many cases, these techniques require WGS, which is still not rapid and economical in the clinical setting.

The goal of this study was to develop a rapid, inexpensive, amplicon sequencing (AmpSeq) molecular method to characterize *E. coli* directly from clinical samples. Other studies that have developed AmpSeq approaches for analyzing clinical samples have focused on the amplification, sequencing, and analysis of the 16S rRNA gene (29), which does not provide the level of resolution needed for accurate species (or subspecies) identification and source attribution. Here, our assay includes multiple targets to determine the presence of *E. coli* and genotype strains within a sample. The utility of our AmpSeq assay, called ColiSeq, is demonstrated on clinical UTI samples and validated with a range of *in silico* and *in vitro* methods.

## MATERIALS AND METHODS

### Reference genome downloading and multilocus sequence typing

Thirty-three thousand three hundred nine *Escherichia* genomes were downloaded on 18 April 2023 with the ncbi-genome-download tool (https://github.com/kblin/ncbi-genome-download) from the RefSeq database (30). To identify any mis-annotated genomes, pairwise distances were calculated with MASH v2.3 (31) for each genome against *E. coli* K-12 strain MG1655 (MG1655) (NC_000913.2) (32). Genomes with a MASH distance >0.06 [~94% average nucleotide identity (ANI)] were considered *Escherichia* near neighbors. One thousand one hundred fifty-one assemblies with >500 contigs were filtered from all downstream analyses. The final reference set consisted of 31,630 *E. coli* genomes and 528 near neighbor *Escherichia* genomes.

A representative set of *E. coli* reference genomes was created from the 31,630 genomes with the Assembly Dereplicator tool v0.1.0 (https://github.com/rrwick/Assembly-Dereplicator) using default settings, resulting in a set of 461 reference genomes (Table S1). The sequence type (ST) of these genome assemblies was determined with FastMLST v0.0.15 (33) and the Clermont group (phylogroup) (34) was determined with the ClermonTyping tool (35). A previously dereplicated set of 516 *E. coli* genomes, using the Assembly Dereplicator method at a less stringent threshold, was also used for a subset of analyses (Table S1).

### Reference UPEC genomes from Flagstaff, Arizona

A recent study analyzed >3,000 *E*. *coli* isolates from meat and clinical samples from Flagstaff, Arizona (23). The WGS data from these samples (PRJNA307689, PRJNA407956) were downloaded, assembled with SPAdes v3.15.5 (36), sequence typed with FastMLST, and analyzed in this study to determine if common sequence types were circulating in Flagstaff.

### Single locus informative marker identification and analysis

To generate a WGS reference tree, the set of 516 *E. coli* genomes (Table S1) was aligned against MG1655 with NUCmer v3.1 (37) in NASP v1.2.1 (38) and a maximum likelihood phylogeny was inferred on the concatenated SNP alignment with RAxML v8.2.4 (39). To identify an appropriate molecular marker for genotyping, the MG1655 genome was sliced into 300 nt fragments, overlapping by 50 nt. Each candidate marker (*n* = 92,789) was extracted from the 516 *E. coli* genome set (Table S1) with BLASTN v2.11.0 (40), aligned with MUSCLE v3.8.31 (41), and a phylogeny was inferred on the alignment with FastTree2 v2.1.10 (41, 42). The Robinson-Foulds (RF) (43) distance was calculated between the candidate marker tree and the WGS tree with DendroPy v4.2.0 (44). All of these functions were wrapped with Phylomark v1.6 (45). A region with a low RF value and a larger number of distinguishing SNPs was chosen as the single locus informative marker (SLIM); this region corresponds to positions 1,834,751 through 1,835,050 in *E. coli* MG1655 and corresponds to locus B21_01711 in *E. coli* BL21 and b1754 in MG1655 (SLIMv2). An additional SLIM marker (SLIMv1), designed earlier from a smaller *E. coli* collection with the same methods, was also included in the ColiSeq assay described below to provide additional phylogenetic resolution; the SLIMv1 corresponds to locus_tag b2244 in MG1655.

To build a reference SLIM database, the SLIMv2 was extracted from the set of 31,630 *E. coli* genomes from BLASTn alignments. The complete SLIMv2 alignment was found in 31,560 genome assemblies (99.8% of all genomes). Unique SLIM alleles were identified with mothur v1.44.3 (unique.seqs command) (46) resulting in 333 unique SLIM variants. In some cases, SLIM variants represent multiple STs and genomes within some STs can belong to multiple SLIM variants (Table S2).

### Minimum spanning set identification

Reference *E. coli* genomes (*n* = 31,630) were aligned against *E. coli* MG1655 with NASP. All SNPs were processed with VaST (47) in order to identify the minimum number of SNPs that provide maximum strain-level resolution. Ten SNPs were identified that provided >90% strain resolution; this set of targets represents the minimal spanning set (MSS) (Fig. S1). For primer design, 125 nt flanking regions in the MG1655 genome were extracted from both sides of each MSS SNP in the reference genome.

### Unique *E. coli* species gene marker

The pan-genome of a reference set of *E. coli* genomes (*n* = 461, Table S1) was identified with LS-BSR v1.2.3 (48). The core genes, defined as having a blast score ratio (BSR) (49) ≥0.8 in all genomes, within these *E. coli* genomes were screened against near neighbor *Escherichia* genomes with LS-BSR. Genes with a BSR value <0.4 in the near neighbor genomes were identified, resulting in three potential candidates for an *E. coli* presence/absence marker. One of these regions was moved forward into the ColiSeq assay; this region corresponds to *yfdX* (B21_02246), a protein of unknown function.

### Primer design and *in silico* optimization

Primer3 v2.3.6 (50) was used to identify candidate forward and reverse primers for the following targets: *E. coli* species marker, 2 SLIMs (v1 and v2), and 10 MSS targets. Primers were selected if they had a predicted amplicon size of between 150 and 300 nucleotides and the melting temperature was between 52°C and 62°C [calculated with IDT OligoAnlyzer Tool (51) using the qpcr specsheet settings]. Predicted amplicons were extracted from alignments with BLASTn, aligned with MUSCLE, and visualized with JalView (52). Based on the alignment, degeneracies were manually added to the primer sequences where needed for maximum genome inclusion.

The primers were then quality checked with Primacy (https://github.com/FofanovLab/Primacy) to limit potential negative primer interactions. The oligos were synthesized with forward (UT1: ACCCAACTGAATGGAGC) and reverse (UT2: ACGCACTTGACTTGTCTTC) universal tails (53) to facilitate index ligation prior to pooling for multiplexed sequencing.

### *In silico* PCR screen

To test for sensitivity and specificity of ColiSeq *in silico*, PCR primers were screened against *Escherichia* genomes with the -search_pcr method in USEARCH v11.0.667 (54), using the following parameters: “-strand both -maxdiffs 2 -minamp 70 -maxamp 1500.” The number and composition of predicted amplicons were manually verified.

### *WG-FAST* validation on SNPs in the ColiSeq data set

The whole genome focused array SNP typing (*WG-FAST*) method genotypes bacterial strains using a subset of all core genome SNPs (55). To demonstrate the ability to genotype *E. coli* strains using SNPs in ColiSeq amplicons, a reference data set was created from a set of 21 characterized *Escherichia* genomes (56). SNPs were identified for all genomes against MG1655 with NASP and a maximum likelihood phylogeny was created from the concatenated SNP alignment with RAxML v8.2.4 (39). Reads from *E. coli* C227-11 (SRR341580), an outbreak strain isolated in 2011 from Europe (57), were aligned with minimap2 v2.24 (58) against ColiSeq amplicons and aligned reads were extracted from the resulting BAM file (*n* = 2,437 paired end reads) with SAMtools v1.9 (59). Mapped reads from C227-11 were inserted into the phylogeny with *WG-FAST* v1.2 with SNPs called at a minimum coverage of 3× to determine if the phylogenetic placement of the genome was consistent with previous results (57).

### Multi-locus sequence typing locus analysis

To compare the number of polymorphisms in ColiSeq regions with traditional markers, seven genes from the PubMLST typing system for *E. coli* (60) were extracted from a set of BLASTn alignments with a custom script (https://gist.github.com/jasonsahl/2a232947a3578283f54c). The number of SNPs contained in a concatenated alignment of MLST markers was calculated with snp-dists v0.8.2 (https://github.com/tseemann/snp-dists). A phylogeny was inferred on the concatenated MLST alignment with raxml-ng v1.1.0 (61) using the GTR + G substitution model; the unweighted Robinson-Foulds distance between this phylogeny and phylogenies inferred on the core genome alignment and the ColiSeq alignment were calculated with the treecompare method in DendroPy.

### Sample collection

Clinical urine samples (*n* = 294) were collected from Flagstaff Medical Center (FMC) from October 2017 to September 2018 under IRB protocol No. 764034-NAH. These samples were collected from patients with urinary tract infections that were confirmed at FMC by culture-based approaches in the clinical laboratory; all samples were transferred to NAU with any identifying patient data removed. Samples were stored at −80°C until processed and precautions were taken to avoid freeze-thaw cycles to prevent any changes in the microbial composition of the samples.

### Urine DNA extraction and validation

Whole DNA was extracted from each urine sample using the Norgen Urine DNA Isolation Kit (Slurry Format) (Norgen Botek Corp Cat# 48800) with two elutions of 100 µL. Of the 294 urine samples that were collected, only 230 of them were extracted (Table S3); the remaining samples did not have enough volume of urine to go through the extraction process. Following extraction, an *E. coli* species-specific PCR assay (described above, Table 1) was run on DNA to confirm the presence of *E. coli*. Five hundred microliters of urine was also plated using a sterile hockey stick on both Luria-Bertani and MacConkey agar and incubated for 72 h at 37°C. For a subset of samples that underwent WGS, single colonies were isolated, DNA was extracted with GenElute (MilliporeSigma, St. Louis, MO), and cultures were frozen at −80°C in 20% (vol/vol) glycerol (Table S3).

**TABLE 1:** Assay information and sequence

Type of target	Locus	Primer	Primer (5′−3′)	Melting temp	Amplicon length	Amplicon length + UT	Position in Amplicon	Reference	Variant	Sequence ID	Start	Stop	*E. coli* hits (*n* = 31630)	NN hits (*n* = 528)
*E. coli* species	B21_02246	Forward	GAGCGTATCTCTGAACAAGG	59.7	110	146	N/A	N/A	N/A	CP032667.1	1389110	1389219	31485	0
		Reverse	GAAGCTTCRYTGGTCAGTTC	60.8										
MSS - SNP	B21_00966	Forward	CAGCATAAAAGAGGAGGATG	57.9	150	186	49	A	G	CP032667.1	2863461	2863312	31582	46
		Reverse	CTGGTTCCKGATACCGATAG	59.9										
MSS - SNP	B21_01903	Forward	CATTCGACTCCGGTTKATTA	58.5	147	183	37	T	C	CP032667.1	1797701	1797555	24981	163
		Reverse	GAAGAAAGCTCAATGGTTCT	58.2										
MSS - SNP	B21_01907	Forward	CGYGAATCAAAATACATCAT	55.8	176	212	114	G	A	CP032667.1	1792511	1792336	31593	528
		Reverse	TTCAGTTTTTCCATNGTTTC	55.9										
MSS - SNP	B21_01912	Forward	TAGATTTAATYRACGGSACB	57.3	181	217	120	C	T	CP032667.1	1788231	1788051	31047	505
		Reverse	TTAATCARYRGGATTTGA	52.4										
MSS - SNP	B21_01973	Forward	AGATCCAGACYAAYGAYGAR	59.6	214	250	113	A	G,T	CP032667.1	1724304	1724091	31584	525
		Reverse	CTGTCWACCGTCTGRATWAT	58										
MSS - SNP	B21_03426	Forward	CATCACATCGCAGTATTCAT	58.1	235	271	121	G	A	CP032667.1	91517	91283	31602	527
		Reverse	CAACAACGATATGCAKGAG	57.4										
MSS - SNP	B21_03444	Forward	AAGATGRGYCAGMACRTTRT	60.7	193	229	88	G	A	CP032667.1	72607	72415	31630	434
		Reverse	GGGCTGGATTATCATTCATT	58										
MSS - SNP	B21_03573	Forward	GCTTTGYTGCAGACCATC	60	182	218	124	C	T	CP032667.1	4594237	4594056	31604	374
		Reverse	TGATTGTGAAATACDGTGAA	56.3										
MSS - SNP	B21_04060	Forward	ATCTAYAAAGCGATGATKGA	56.9	172	208	66	T	G,C,A	CP032667.1	4072460	4072289	31605	527
		Reverse	AYTCTTCTTTTGCGGTATCA	58.7										
MSS - SNP	B21_04066	Forward	AGTTTATGGAACARYACCAC	58	144	180	75	G	A	CP032667.1	4065665	4065522	31615	527
		Reverse	CCAGCGTATTAARATGGAAG	57.4										
SLIM v1	B21_02129	Forward	TGTCATTAGTGAAGCCTCTA	58.1	262	298	N/A	N/A	N/A	CP032667.1	1525852	1526113	31575	517
		Reverse	TTGCTGTTTTATCATGGC	56										
SLIM v2	B21_01711	Forward	MTTGTGGCAATWTCTTGAYG	58.3	279	315	N/A	N/A	N/A	CP032667.1	2045846	2045568	31562	165
		Reverse	AARTTGGCRACHACCTTCG	61.4										

### ColiSeq amplification and sequencing of clinical samples

Two different PCR master mixes—Promega PCR Master Mix (Promega Corporation, Madison, Wisconsin, United States) and KAPA2G Fast Multiplex PCR Mastermix (Roche, Indianapolis, IN)—were evaluated for the amplicon sequencing multiplex PCR; the master mix with the strongest amplification band was selected on a per sample basis. Promega/Kapa2G 2× pre-mixed master mixes were used in a final 1× concentration in the final PCR and water was added to bring the volume of each reaction to 22.5 µL prior to adding 2.5 µL of template DNA. The PCR conditions consisted of an initial denaturation at 95°C for 3 min, followed by 30 cycles of denaturation at 95°C for 15 s, annealing at 65°C for 30 s, and extension at 72°C for 90 s.

The PCR product underwent two 1.5× AmpPure bead (Beckman Coulter Cat# A63880) purifications, consecutively. The bead-cleaned products were then indexed with 8 bp indices in a second PCR amplification. 2× Kapa HiFi HotStart ReadyMix (Roche, Indianapolis, IN) was diluted to 1× and mixed with 1 µM of forward and reverse indexing oligo. Two microliters of purified PCR product was added, and the reaction was brought to 25 µL with water. Thermocycler conditions included an initial denaturation step of 98°C for 2 min followed by 6 cycles of a 98°C denaturation step for 30 s, a 60°C annealing step for 20 s, and a 72°C extension step for 30 s. These six cycles were followed by a final extension step of 72°C for 5 min.

After indexing, another 1.5× bead purification was performed, and the products were normalized for multiplexed sequencing with the Invitrogen SequalPrep Normalization 96-well plate kit (ThermoFisher Cat# A1051001), following manufacturer’s directions. After the samples were eluted, they were pooled together and a 1× bead clean-up was performed on the pool. When eluting the DNA off the beads with Tris-tween, 1/10th of the initial pool volume was used to elute and concentrate the pool. The final pool was quantified on a Qubit 4 Fluorometer (ThermoFisher Cat# Q33226) and sequenced on the Illumina MiSeq platform.

### Analysis of ColiSeq amplicon sequence variants for clinical samples

Following Illumina sequencing, amplicon sequence variants (ASVs) of the SLIMv2 were identified with QIIME2 v2022.2.0 (62). The exact commands used to process the amplicon data and identify ASVs with QIIME are available for full reproducibility (https://gist.github.com/jasonsahl/bca2067afd954853b25f51803f1aa18c#file-slim_qiime_commands-txt).

### ColiSeq cost estimates

Costs associated with the ColiSeq assay were estimated on a per sample basis (Table S4). The cost estimations are based on a range of sequencing depths and are current to November 2023 prices.

### *WG-FAST* database development and data analysis for reference genomes and clinical samples

Urine samples were genotyped based on a partial set of single-nucleotide polymorphisms in ColiSeq amplicons using *WG-FAST*. The reference set of 461 dereplicated genomes (Table S1), genomes from a recent study of *E. coli* in Flagstaff (*n* = 1,335) (23), and a set of genomes generated as part of this study (*n* = 37, see methods below) were aligned against MG1655 with NUCmer within NASP. A phylogeny was inferred from the concatenated SNP data with FastTree2 v2.1.11 (41, 42). To obtain the targeted set of SNPs from the ColiSeq assay, the NASP matrix was filtered to only include positions present in the ColiSeq amplicons. All clinical ColiSeq data were then placed into the WGS phylogeny with *WG-FAST* at a minimum depth of coverage of 3×, which allows for genotyping at low-coverage loci. Publicly available genome assemblies that were not closely related to urine sample genotypes were removed from the data set, and the *WG-FAST* process was repeated with the following exceptions: the core genome SNP tree was generated with IQ-TREE v2.2.2.3 (63, 64), and both clinical sample data and WGS data were inserted into the phylogeny with *WG-FAST* to determine if the placements of each data type within the tree were consistent.

### Limit of detection for ColiSeq assay

To determine the limit of detection (LOD) of the multiplexed ColiSeq assay, three sample DNAs were quantified on a Qubit 4 Fluorometer (ThermoFisher Cat# Q33226), diluted, amplified with the ColiSeq assay, and sequenced on the Illumina MiSeq platform. Samples included in the LOD analysis include an ST131 isolate (FMC_UTI_02545:SAMN04414795), an ST10 isolate (HS-U-736-I-03:SAMN35335793), and a ST404 isolate (HS-U-877-I-03:SAMN35335903). The number of genomic equivalents was calculated by using the genome assembly size and assuming that the mass of a DNA base pair is 650 daltons. The breadth of coverage was calculated with SAMtools across each amplicon in the multiplex at a minimum depth of 3× and values >80% were considered to be present.

### *In silico* limit of detection for ColiSeq assay

Reads were randomly subsampled from the isolate genome HS-U-000681 (SRR24939606) at a range of depths (100–1,000 paired reads, stepping by 100) with seqtk v1.3 (https://github.com/lh3/seqtk), and the breadth of coverage across amplicons was calculated with SAMtools at 3× depth; the number of subsampled data sets (100 reads) with a breadth >80% was identified and plotted.

### ASAP analysis

The ColiSeq data for clinical samples were also analyzed with the ASAP pipeline (https://github.com/TGenNorth/ASAP) using the “ASAP_assaydetails_tsv.xsl” output transform format (https://github.com/TGenNorth/ASAP/tree/public/output_transforms). This allows for easy visualization of the number of reads and coverage breadth for presence/absence assays. For SNP assays, the number of reads, coverage breadth, SNP position, depth of coverage, reference call, and percentage of calls for each nucleotide at each SNP position were reported. Mixtures/co-infections were identified by >10% minor allele frequency.

### Whole-genome sequencing

For whole-genome sequencing, sample selection was restricted to urine samples that grew red colonies on MacConkey agar. Using ColiSeq data analyzed by *WG-FAST*, a diversity of samples from across the global phylogeny in proximity to other Flagstaff UTI and *E. coli* positive food samples (23) were selected. For these samples, DNA was extracted from a single colony that grew on MacConkey agar at 37°C overnight with GenElute (NA2110-1KT). Approximately 250 ng of DNA was prepared for sequencing on the Illumina MiSeq platform.

### Enterobacterial repetitive intergenic consensus PCR and targeted mixture sequencing

Using the ASAP analysis data, possible mixtures were interrogated further using the enterobacterial repetitive intergenic consensus (ERIC) PCR assay. The ERIC PCR assay amplifies regions between neighboring repetitive elements, which generates DNA fingerprints that enable the differentiation of Enterobacterial species and strains (65). Using the ASAP results, a total of 10 samples were chosen to examine possible mixtures within samples: HS-U-000720, HS-U-000728, HS-U-000855, HS-U-000910, HS-U-000940, HS-U-000942, HS-U-000946, HS-U-000973, HS-U-000765, and HS-U-000962. Five isolation streaks were made for each sample on MacConkey agar plates from MacConkey populations in LB + 20% glycerol stocks. The isolation streaks were incubated at 37°C overnight. After incubation, 20 single colonies were picked using an inoculation needle for each sample and swirled into 15 µL of molecular grade water. Quarter lawns were made for each single colony as well by streaking the single colony on a quarter of a MacConkey agar plate prior to swirling it in the water and incubating the plates at 37°C. The single colonies underwent a boil at 95°C for 10 min, followed by centrifugation at 2,200 rcf for 1 min. The boiled product was then used as input for the ERIC assay with the following protocol: 1× Q5 Hot PCR Master Mix, 1.5 µL gDNA template, and 10 µM ERIC2 and ERIC1R primers. Specific PCR parameters were as follows: initial denaturation at 98°C for 2 min, 35 cycles of denaturation at 98°C for 10 s, annealing at 52°C for 30 s, and extension at 72°C for 90 s, with a final extension at 72°C for 2 min. The PCR product was run on a 1% Lithium Borate agarose gel at 200 V for approximately 1 h using a 1 kb ladder and then viewed with a Bio-rad Gel Doc. Gel images were examined to determine if there were multiple unique banding patterns within a single sample across the 20 colonies. For two samples (HS-U-000946, HS-U-000720) that had multiple banding patterns, each colony that contained a unique pattern underwent a gDNA extraction using the quarter lawns generated in the single colony picking described above and was sequenced on the Illumina MiSeq platform.

## RESULTS

The goal of this study was to develop a method to rapidly and inexpensively detect and genotype *E. coli* directly from clinical samples. We developed a multiplex, amplicon sequencing assay (ColiSeq), guided by comparative genomics, that can be used for high-throughput genotyping of *E. coli* in complex samples. The ColiSeq assay targets a region for determining *E. coli* presence/absence, 2 regions determined to be highly informative for differentiating *E. coli* strains [single locus informative marker (SLIM); SLIMv1 and SLIMv2], and 10 SNP loci that provide maximum resolution of *E. coli* strains [minimal spanning set (MSS)].

### ColiSeq assay development and testing

An *in silico* PCR screen of ColiSeq primers designed in this study (Table 1) against a diverse set of reference genomes demonstrates that MSS and SLIM assays broadly amplify both *E. coli* and near-neighbor species, while the *E. coli* species marker was specific to *E. coli* (Table 1).

To demonstrate that SNPs targeted in the ColiSeq assay can provide accurate genotypic information, a reference set of *E. coli* genome assemblies (*n* = 516) (Table S1) was aligned against ColiSeq target amplicons used in this study and 435 SNPs were identified. The resulting phylogeny demonstrates that many of the primary phylogroups were recovered, and the amplicon tree was generally consistent with the core genome phylogeny (Fig. S2).

**Fig 1:**
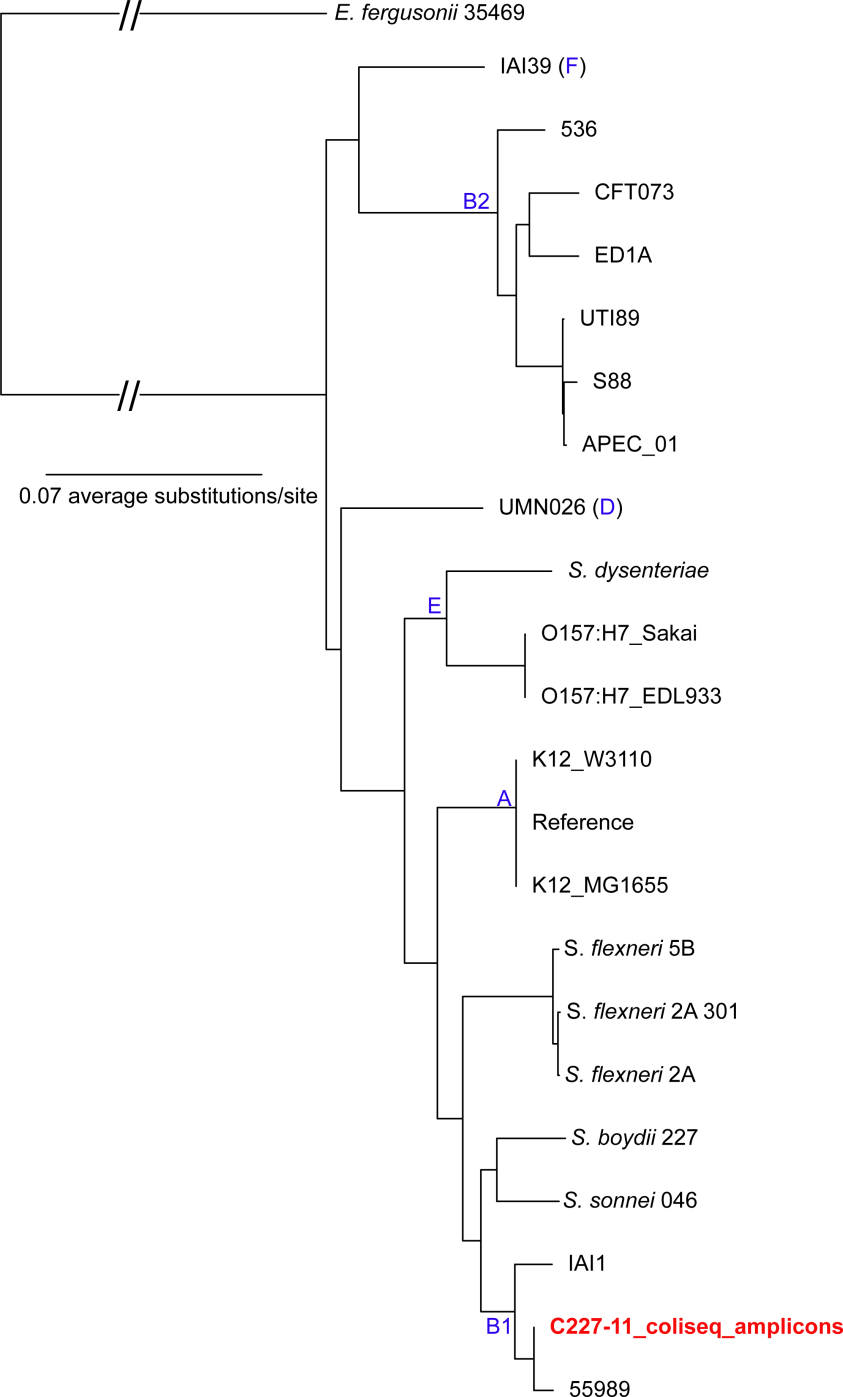
The placement of sample C227-11 (57) (shown in red), only including ColiSeq positions, into a maximum-likelihood phylogeny of reference *E. coli* genomes (56). SNPs were identified by NASP (38) and the reference genome was MG1655 (accession: NC_000913.2). Phylogroups are labeled in blue.

To demonstrate the ability of the ColiSeq amplicons to provide strain level resolution, the C227-11 STEC genome was downloaded, aligned to ColiSeq amplicons, and placed into an existing phylogeny of reference genomes (56) with *WG-FAST*. The results demonstrate that C227-11 grouped in phylogroup B1 with *E. coli* 55989 (Fig. 1), a result that is consistent with the whole-genome analysis (57) and demonstrates the power of SNPs in the ColiSeq amplicons to accurate type strains.

### Comparison of ColiSeq with MLST

For the pubMLST system, seven genes are typically amplified with PCR and Sanger sequenced; a concatenation of genes extracted from genomes analyzed in this study (*n* = 461) consists of 3,423 nucleotides and 602 SNPs. The ColiSeq system includes 2,445 nucleotides and 691 SNPs from the same set of genomes. A comparison of phylogenies with the unweighted Robinson-Foulds (RF) metric demonstrated that the ColiSeq phylogeny was more similar to the whole-genome phylogeny (RF = 878) compared to the concatenated MLST phylogeny (RF = 1838).

### Amplicon sequence coverage of ColiSeq data across clinical urine samples

Of the 294 clinical urine samples that were collected, adequate DNA was extracted and analyzed from 230 samples (Table S3). After PCR amplification with the ColiSeq multiplex assay and sequencing on the Illumina MiSeq platform, the breadth of coverage (minimum 3×) of all clinical data sets across the predicted amplicons demonstrated that broad coverage was generally observed across amplicons (Table S5). One amplicon, B21_01903, showed low breadth of coverage in 29 urine samples; an *in silico* screen across >31,000 reference *E. coli* genomes (Table 1) also demonstrated that many isolate DNAs would not amplify with the B21_01903 primers designed in this study (Table 1). An investigation into genomes that would be missed with this assay revealed that 85% of genomes (*n* = 5,002) from phylogroup B2 would not amplify with this primer set; the majority of UPEC fall within this phylogroup (21). This target could be removed from the multiplex assay but could also be included to provide additional resolution in genomes that contain this target.

**Fig 2:**
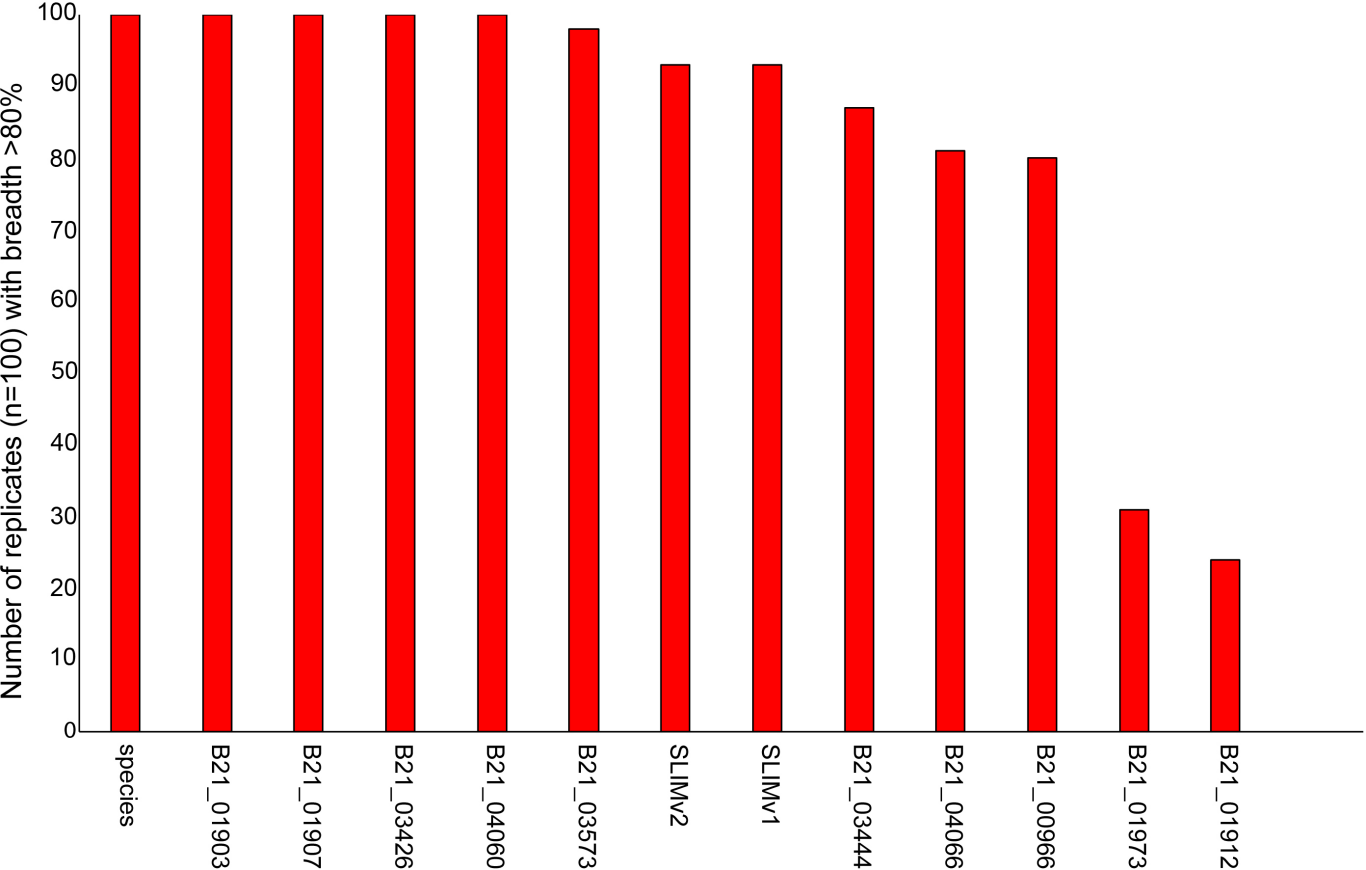
The number of randomly sampled data sets (of 100), at 100 reads, that have a breadth of coverage greater than 80% (at 3× depth) across ColiSeq amplicons.

Amplified samples in this study were sequenced to a high depth (e.g., ~85,000 paired end reads per sample). However, a sub-sampling test demonstrated that as few as 100 randomly selected reads can provide coverage across many amplicon targets, including one that is unique to *E. coli* (Fig. 2). The cost of processing a single sample with the ColiSeq assay was estimated between $20 and $28 depending on sequencing depth (Table S4).

### Single locus informative marker performance and mixture detection

The SLIMv2 variants were identified with QIIME2 across all clinical urine samples, resulting in 30 amplicon sequence variants (ASV) (Table S6) called across 219 samples (Table S7); 11 of the 230 samples did not have enough coverage across the SLIMv2 amplicon to facilitate genotyping based on the QIIME2 ASV approach. An analysis based on extracting the SLIMv2 from reference *E. coli* genomes demonstrated that many SLIM alleles were identical across multiple genotypes (Table S2), suggesting limited resolution within those alleles.

For 11 samples (HS-U-000946, HS-U-000727, HS-U-000770, HS-U-000810, HS-U-000855, HS-U-000868, HS-U-000878, HS-U-000682, HS-U-000699, HS-U-000720, HS-U-000940), multiple SLIM ASVs were identified (Table S7), suggesting a mixture of *E. coli* genotypes. An analysis with ASAP across multiple loci (MSS SNP sites) confirmed mixtures for six of these samples (Table S8); the discordance between methods is not surprising as the ASAP output only includes a single nucleotide locus per amplicon and the SLIM ASV includes the entire amplicon. ASAP also identified mixtures in two additional samples (HS-U-000942, HS-U-000973), demonstrating the power of incorporating additional loci when analyzing mixed samples. To confirm mixtures based on ASAP results, an analysis was performed using ERIC PCR and multiple genotypes were observed for all samples; for two of these samples (HS-U-000720, HS-U_000946), multiple whole genomes were generated and separate genotypes were identified (Table S3).

Among the 219 urine samples processed with QIIME2, the most common SLIM ASV (4bebf4b720a328bb27991c67bdd0c3a2) was associated with an allele representing >160 STs (SLIM variant 4 in Table S2), which obscures definitive genotyping. The second most common SLIM ASV in the clinical urine sample data set (5e070755c3418ef0eaf26ce443097dd0) was associated with a ST that includes ST131 (SLIMv2 variant 7; Table S2), a sequence type commonly observed in UTI samples (66).

### Comparing genotype resolution between ColiSeq and WGS using *WG-FAST*

**Fig 3:**
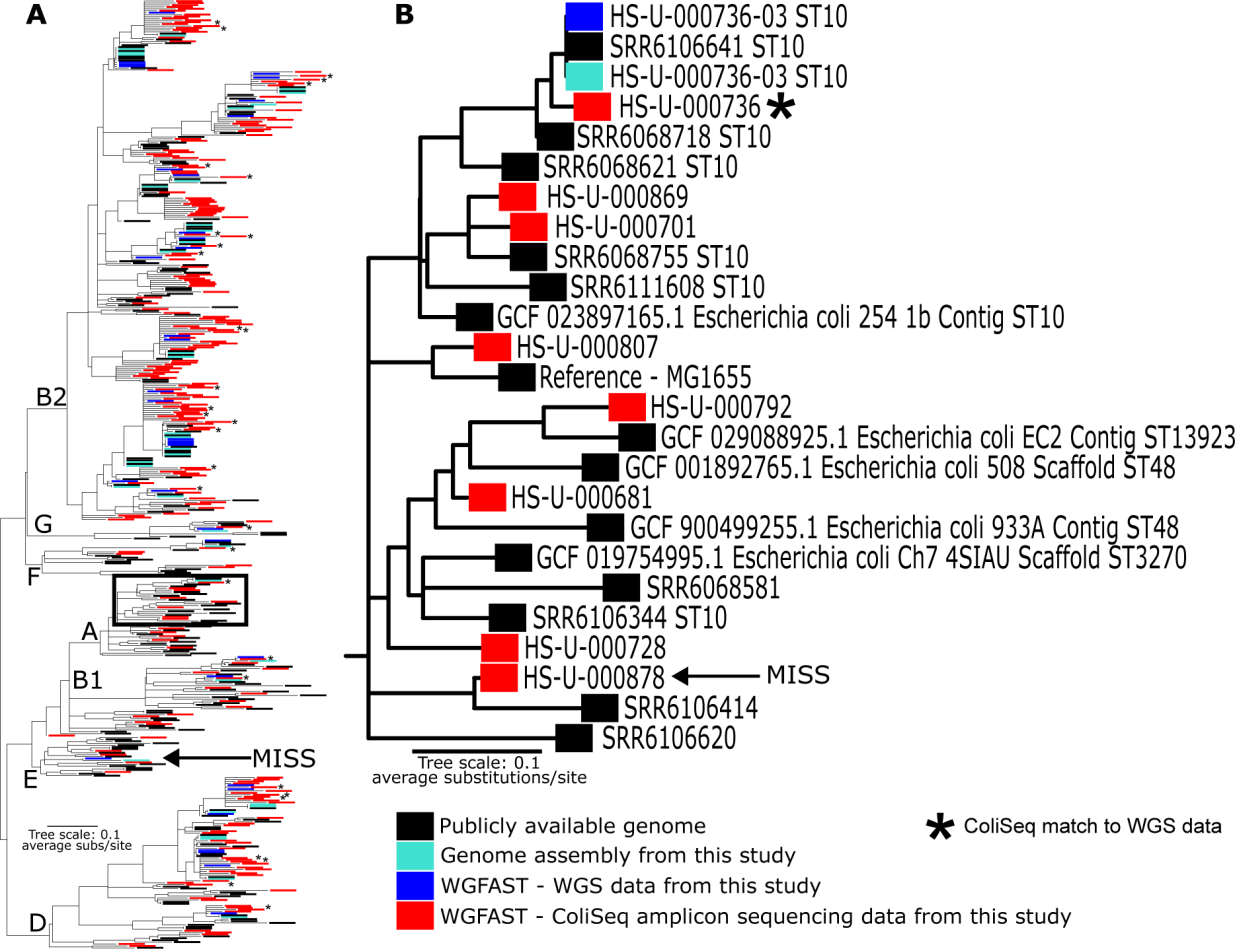
A maximum-likelihood phylogeny including reference genomes as well as samples amplified with ColiSeq (inserted into phylogeny with *WG-FAST*). Panel A demonstrates the placement of 230 clinical urine samples (red) within a core genome SNP phylogeny of *E. coli* isolates. The phylogeny includes whole-genome sequenced isolates from 36 of the clinical urine samples (teal; WGS, blue; *WG-FAST* using WGS data) to determine if ColiSeq genotyping is consistent with WGS data. Stars indicate ColiSeq genotypes matching WGS data. Phylogroups are labeled in black. Panel B corresponds to the black box in panel A. A clinical sample (HS-U-000736) with a ColiSeq genotype matching WGS data and a clinical sample (HS-U-000878) with a ColiSeq genotype inconsistent with WGS data are shown. Arrows indicate the ColiSeq data and WGS data for sample HS-U-000878.

To demonstrate the potential of ColiSeq to provide strain-level resolution on clinical samples, 36 samples with paired WGS and AmpSeq data were processed with *WG-FAST*; only the core genome of K12 MG1655 was used for genotyping, which limits the amount of resolution available from the entire *E. coli* pan-genome. The results demonstrate that 32 ColiSeq data sets were consistent with the WGS data, 1 was a near miss, and 3 samples were total misses (Fig. 3; Fig. S3; Table S3). An example of a clinical sample with a genotype matching WGS data and an example of a sample with a genotype inconsistent with WGS data are provided in panel B of Fig. 3. Sample HS-U-000720 was a confirmed mixture for which two isolates were whole-genome sequenced. Although mixed genotypes have been shown to confound accurate phylogenetic placement with *WG-FAST* (55), especially when present in nearly equal proportions (e.g., 60/40), this sample is appropriately genotyped as one of the isolates sequenced from this sample (Figure S3). A lack of coverage across reference positions and mixtures of multiple strains are two demonstrated explanations for the relatively poor genotyping placement for a subset of samples.

All additional urine samples processed in this study were placed into the phylogeny with *WG-FAST* (*WG-FAST* failed to place sample HS-U-000866 into the phylogeny due to low coverage and a lack of distinguishing SNP calls). The ST for the closest match was recorded (Table S3) based on the phylogeny (Figure S3); in some cases, the matching ST could not be determined based on the phylogeny. For clinical samples where the putative ST was derived from the phylogenetic placement, the dominant STs were ST131 and ST69; other common STs were ST12, ST95, and ST73. Sixteen clinical samples were placed within a clade that includes ST14, ST404, and ST1193. Frequently identified sequence types in this study overlap with the most common STs in human cases (ST131, ST95, ST73, and ST69) identified in a recently published study (23) that sampled *E. coli* from meat and UTIs in Flagstaff several years prior to the sampling conducted as part of this study.

### ColiSeq limit of detection

The *E. coli* species marker was consistently identified in each dilution down to ~2 genome equivalent (GE) per milliliter (Table S9); these metrics were based on breadth of coverage at a minimum depth of 3×. For the MSS targets, a 1:1,000 dilution (~170 GEs per µL) generally returned enough amplification to call positives and accurately genotype UPEC.

### Whole-genome sequencing of select isolates

Based on the ColiSeq analysis of urine samples compared to previous UTI samples obtained from Flagstaff, 40 isolates representing the diversity of observed strains and potential mixtures were obtained from clinical urines and whole genome sequenced. For sequenced isolates, 20 unique STs were identified (Table S3), including two novel STs (ST11973, ST11974); the most common ST was ST131 (*n* = 8). For two samples (HS-U-000720 and HS-U-000946), multiple genomes were sequenced and confirmed the presence of multiple STs within the sample (Table S3).

## DISCUSSION

In this study, we describe the development and validation of a multiplexed amplicon sequencing assay known as ColiSeq to amplify and genotype *Escherichia coli* directly from clinical samples. Although all validation in this study was performed on UTI samples, ColiSeq should work on other sample types, including clinical diarrheal samples, although additional validation would be needed, including with deconvolution of mixed strains in stool. The ColiSeq assay consists of 13 amplicons designed to provide genotyping in a rapid, inexpensive, and highly multiplexed assay. The system is dynamic, and new amplicon targets [e.g., AMR markers, informative mobile genetic elements (23), or pathovar-specific markers] can be easily added as needed.

*In silico* experimentation demonstrated the utility of ColiSeq for genotyping *E. coli*. We successfully placed C227-11, a strain associated with an *E. coli* outbreak in 2011 (67), into a phylogeny of reference genomes (56), and the results were highly consistent with genotyping using the complete genome (57) (Fig. 1). The ColiSeq assay targets contain enough polymorphic information to infer a phylogeny that is generally congruent with the core genome phylogeny (Figure S2). Although other sub-genomic methods, such as multi-locus sequence typing, have been used for genotyping from urine (68), the sequence type alone cannot confirm transmission, and a phylogeny inferred from the concatenated MLST alignment can be highly discordant with whole genome phylogenetics (45). This incongruence is demonstrated in the analysis of a representative set of *E. coli* genomes, where B2 generally groups together across methods, while phylogroup A is more dispersed throughout the phylogeny using MLST markers (Figure S2). Sub-genomic methods, including MLST and ColiSeq, can be used to exclude transmission events through the identification of divergent genotypes and reduce the need for WGS efforts to interrogate the whole genome in depth. Additionally, ColiSeq is easily sequenced on high-throughput sequencing platforms, like Illumina, whereas MLST provides fewer discriminatory SNPs and needs to be sequenced with longer reads (e.g., 300 nts), to recover the larger amplicons.

We validated the ColiSeq assay on 230 clinical urine samples to show that it can accurately genotype *E. coli* directly from clinical specimens. We built a phylogeny using amplicon sequences from the ColiSeq assay, a set of reference genomes, whole genomes generated as part of this study, and a set of genomes analyzed from urine and food samples from Flagstaff (23). The placement of ColiSeq amplified samples was consistent with corresponding WGS samples (Fig. 3), indicating the potential for using sub-genomic data in source attribution applications. Additionally, many samples were placed within clades with the major pandemic UPEC clones (Fig. S3); these include ST131, ST73, ST69, and ST95 (69). These same STs were found previously in urine and food samples from Flagstaff (23), indicating that these genotypes are circulating in the Flagstaff community and are temporally stable.

Knowledge of strain mixtures within a sample can be important when monitoring recurrent UTIs or considering treatment options as multidrug-resistant UPEC strains could defy some antibiotic regimens. Strain mixtures of multiple genotypes were identified directly from urine samples using the ColiSeq assay. To address the difficulties of identifying strain mixtures within samples, we identified single locus informative markers (SLIMv1 and SLIMv2) that can be evaluated in conjunction with MSS loci for mixture deconvolution. The SLIMv2 analysis identified mixtures in 11 samples, with 6 of these confirmed by orthogonal approaches including ASAP and ERIC. Additionally, the SLIMs and 9 of the 10 MSS loci are widely conserved across known *E. coli* genomes (Table 1), suggesting that they could be used to compare *E. coli* across a range of complex samples, which could include not only clinical samples but other sample types as well (e.g., wastewater, agricultural or environmental). For some *E. coli* genotypes, the SLIM offers only broad genotyping resolution, while for other genotypes, the SLIM designation is likely to be highly specific. When the SLIM is combined with additional loci, the genotyping resolution improves which could allow for surveying *E. coli* in complex backgrounds.

As part of this work, we identified an *E. coli* species marker that is highly conserved in the species and is absent from near neighbor *Escherichia* genomes, including known cryptic species (70); this marker is associated with the *yfdX* gene, which has no known function (71). In a limit of detection (LOD) analysis, the *E. coli* species marker assay could detect the pathogen directly from urine down to <2 genomic equivalents (GE) per milliliter (Table S9), which could be effective at detecting even very early infections. For other ColiSeq targets, ~1.7 × 10^5^ GEs/mL would result in detection as well as genotyping, which is consistent with the concentration of *E. coli* in confirmed UTIs (72). The assay should also be applicable to other sample types with similar or higher *E. coli* loads (e.g., stool samples, wastewater). Sub-sampling reads from a known sample demonstrated that as few as 100 paired reads could provide accurate genotyping (Fig. 2), potentially allowing thousands of samples to be efficiently and inexpensively combined on a single sequencing run.

The direct detection of *E. coli* from clinical samples, including urine, can produce rapid, actionable results without the need to culture. Recently, work has demonstrated the power of linking UTI isolates with zoonotic UPEC found in food (23), suggesting that ColiSeq could serve as a powerful tool to genotype directly from specimens, guiding contact tracing and source attribution efforts by identifying potential *E. coli* transmission events without the need to culture and whole-genome sequence the pathogen. Once potential matches are identified with ColiSeq; WGS and pan-genome analyses could then identify transmission and outbreak tracing by taking maximum advantage of the known diversity of *E. coli*. The ability to deconvolute mixtures and perform high-resolution genotyping will also help monitor recurrent UTIs (73), which may help guide treatment decisions. As sequence-based clinical diagnostics become more mainstream, ColiSeq will represent a powerful tool to monitor clinical UTIs and focus intervention, surveillance, and decontamination efforts to reduce the frequency of transmission events.

## Data Availability

Sequence data generated as part of this study were deposited under BioProject PRJNA853792.

## SUPPLEMENTAL MATERIAL

Fig. S1 spectrum.04139-23-s0001.pdf Saturation curve for minimal spanning set analysis.

Fig. S2 spectrum.04139-23-s0002.pdf Comparison of phylogenies inferred from different data types.

Fig. S3 spectrum.04139-23-s0003.pdf A tree showing the results of a WG-FAST analysis.

Supplemental tables spectrum.04139-23-s0004.xlsx Tables S1 to S9.

ASM does not own the copyrights to Supplemental Material that may be linked to, or accessed through, an article. The authors have granted ASM a non-exclusive, world-wide license to publish the Supplemental Material files. Please contact the corresponding author directly for reuse.
