# Supplementary material for: ColiSeq: a multiplex amplicon assay that provides strain level resolution of Escherichia coli directly from clinical specimens
Source: Microbiol Spectr. 2024 Apr 23;12(6):e04139-23. doi: 10.1128/spectrum.04139-23 (PMC11237721; doi:10.1128/spectrum.04139-23)
Supplement: Fig. S1 — Saturation curve for minimal spanning set analysis. [file spectrum.04139-23-s0001.pdf]

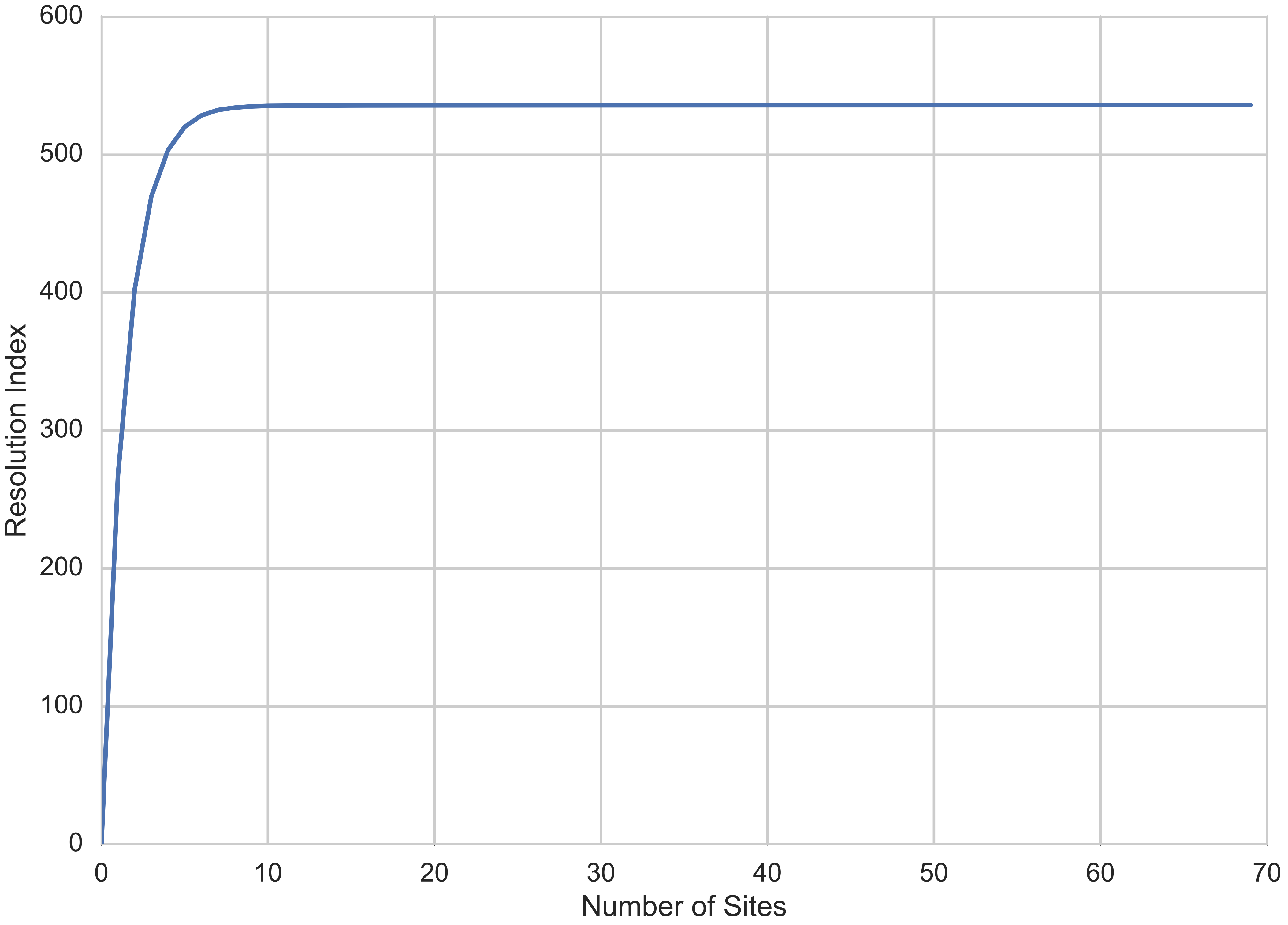

Figure S1: A curve showing the number of genomic sites required for full phylogenetic resolution using VaST (47). Ten genomic sites yield a high resolution index (number of analyzed genomes minus the average unresolved group size (47)) and were selected as MSS targets.
