# Supplementary material for: ColiSeq: a multiplex amplicon assay that provides strain level resolution of Escherichia coli directly from clinical specimens
Source: Microbiol Spectr. 2024 Apr 23;12(6):e04139-23. doi: 10.1128/spectrum.04139-23 (PMC11237721; doi:10.1128/spectrum.04139-23)
Supplement: Fig. S2 — Comparison of phylogenies inferred from different data types. [file spectrum.04139-23-s0002.pdf]

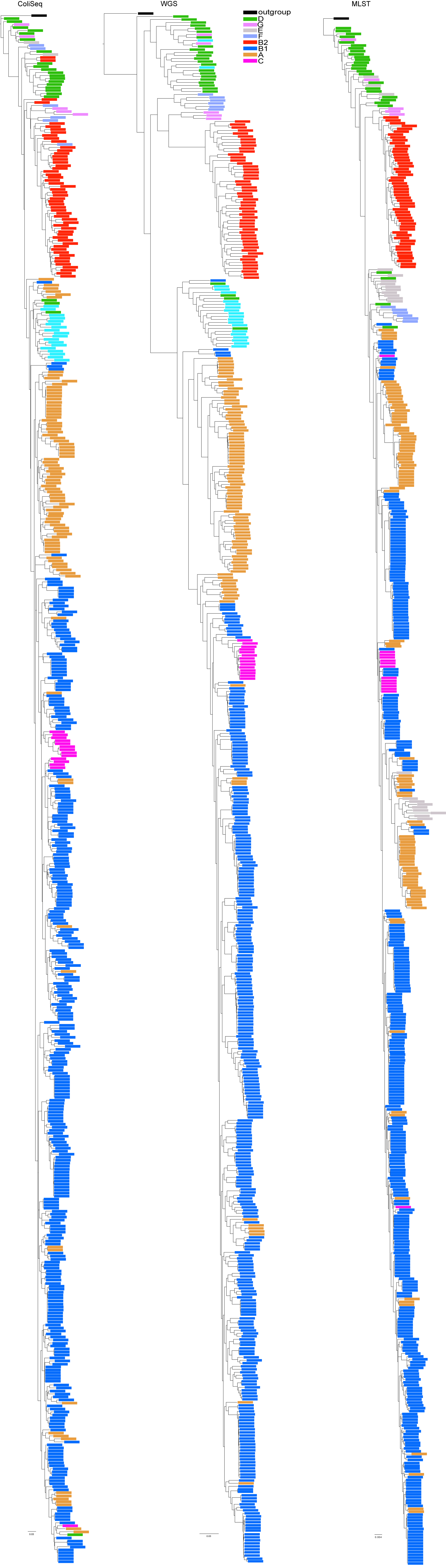

Figure S2: Phylogenies inferred from amplicon (left), WGS (middle) and MLST (right) data. The amplicon phylogeny is generally congruent with the WGS phylogeny while the MLST phylogeny demonstrates mixtures of phylogroups. Phylogroups are denoted with different colors.
