# Supplementary material for: ColiSeq: a multiplex amplicon assay that provides strain level resolution of Escherichia coli directly from clinical specimens
Source: Microbiol Spectr. 2024 Apr 23;12(6):e04139-23. doi: 10.1128/spectrum.04139-23 (PMC11237721; doi:10.1128/spectrum.04139-23)
Supplement: Fig. S3 — A tree showing the results of a WG-FAST analysis. [file spectrum.04139-23-s0003.pdf]

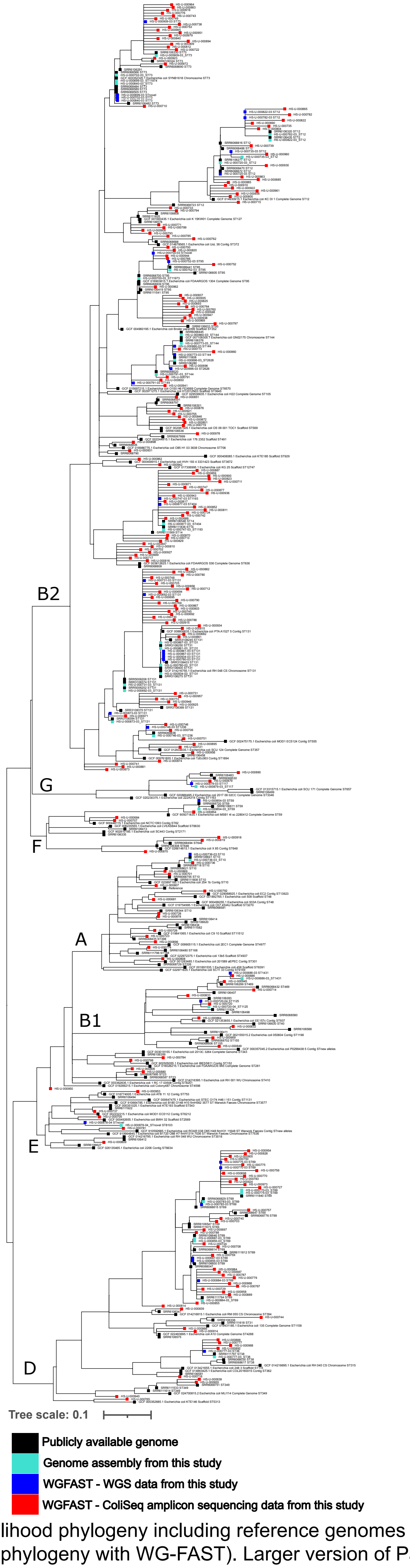

Figure S3: A maximum-likelihood phylogeny including reference genomes as well as samples amplified with ColiSeq (inserted into phylogeny with WG-FAST). Larger version of Panel A from Figure 3. The phylogeny includes whole genome sequenced isolates from 36 of the clinical urine samples (teal; WGS, blue; WG-FAST using WGS data) to determine if ColiSeq genotyping is consistent with WGS data. Stars indicate ColiSeq genotypes matching WGS data. Phylogroups are labeled in black.
